# Large hemangiopericytoma of the pelvis—towards a multidisciplinary approach

**DOI:** 10.1186/s12957-015-0675-6

**Published:** 2015-08-28

**Authors:** Mohammad Fard-Aghaie, Gregor A. Stavrou, Human Honarpisheh, Klaus J. Niehaus, Karl J. Oldhafer

**Affiliations:** Department of General and Abdominal Surgery, Asklepios Hospital Barmbek, Ruebenkamp 220, 22291 Hamburg, Germany; Semmelweis University Budapest, Campus Hamburg, Hamburg, Germany

**Keywords:** Hemangiopericytoma, Solid fibrous tumor, Arterial embolization, Pelvic tumor

## Abstract

**Background:**

In 1942, Stout described tumors which derive from Zimmerman’s pericytes and suggested the term hemangiopericytoma (HPC). These tumors, which are often highly vascularized, pose difficulties in the surgical management regarding blood loss and complete resection. Therefore, preoperative management seems to be an essential part in dealing with these issues.

**Case presentation:**

We present a 70-year-old female patient with a large HPC in the pelvis. Preoperative embolization of the tumor was performed, and 2 weeks after the intervention, we completely resected the tumor with minimal blood loss.

**Conclusion:**

In which cases do we need preoperative treatment, especially emboliziation of hemangiopericytomas/solid fibrous tumors (SFT)? Although preoperative embolizations of tumors are now commonly undertaken, as for now, neither a clear statement nor a standardized approach has been given or developed. The purpose of this article is to provide our experience with preoperative embolization and to start a new discussion concerning a standardized approach.

## Background

Hemangiopericytomas are tumors which derive from Zimmerman’s pericytes. Stout provided the first report and established the term hemangiopericytoma (HPC) [1]. These are now reclassified as solid fibrous tumors. A surgical therapy should involve a multimodal approach, as primary surgery can cause severe blood loss in these highly vascularized tumors. Diagnostic tools like biopsies should be conducted with great caution, if HPCs are considered, as these tumors can lead to exsanguination [2].

In 1976, Smith et al. described the first case of preoperative embolization of retroperitoneal hemangiopericytomas [3]. Although the resection was successfully undertaken, after this experience, only a handful cases were described which advocated a preoperative treatment prior to surgery. Smullens et al. showed in their report of two patients that a debulking of HPCs, especially concerning the massive blood loss, can be eased using preoperative embolization [4]. In 2000, Morandi et al. published a report of a patient, who was diagnosed with a massive tumor in the mediastinum. Radiological finding suggested a resectable mass. The patient underwent right thoracotomy, but massive bleeding led to the abortion of the procedure and only a biopsy was obtained. The patient lost 2.1 L of blood peroperatively. Subsequently, an arterial embolization was performed. Ten days later, the tumor was resected with minimal blood loss. Therefore, the authors advocated a preoperative embolization of highly vascularized HPCs [5]. Another case of a lipomatous hemangiopericytoma, described by Dozois et al., showed that preoperative embolization, although as described in this case, not as successful as desired, was crucial to perform the debulking [6].

These cases show that in highly vascularized tumors, especially in HPCs, preoperative arteographic imaging and subsequent embolization, even if only performed partly, reduce preoperative blood loss and increase the chance to perform a radical resection.

The purpose of this article is to begin a new discussion concerning the approach to highly vascularized tumors, especially hemangiopericytomas. As to this date, a standardized approach, which takes these aforementioned aspects into account, does not exist.

## Case presentation

**Fig. 1:**
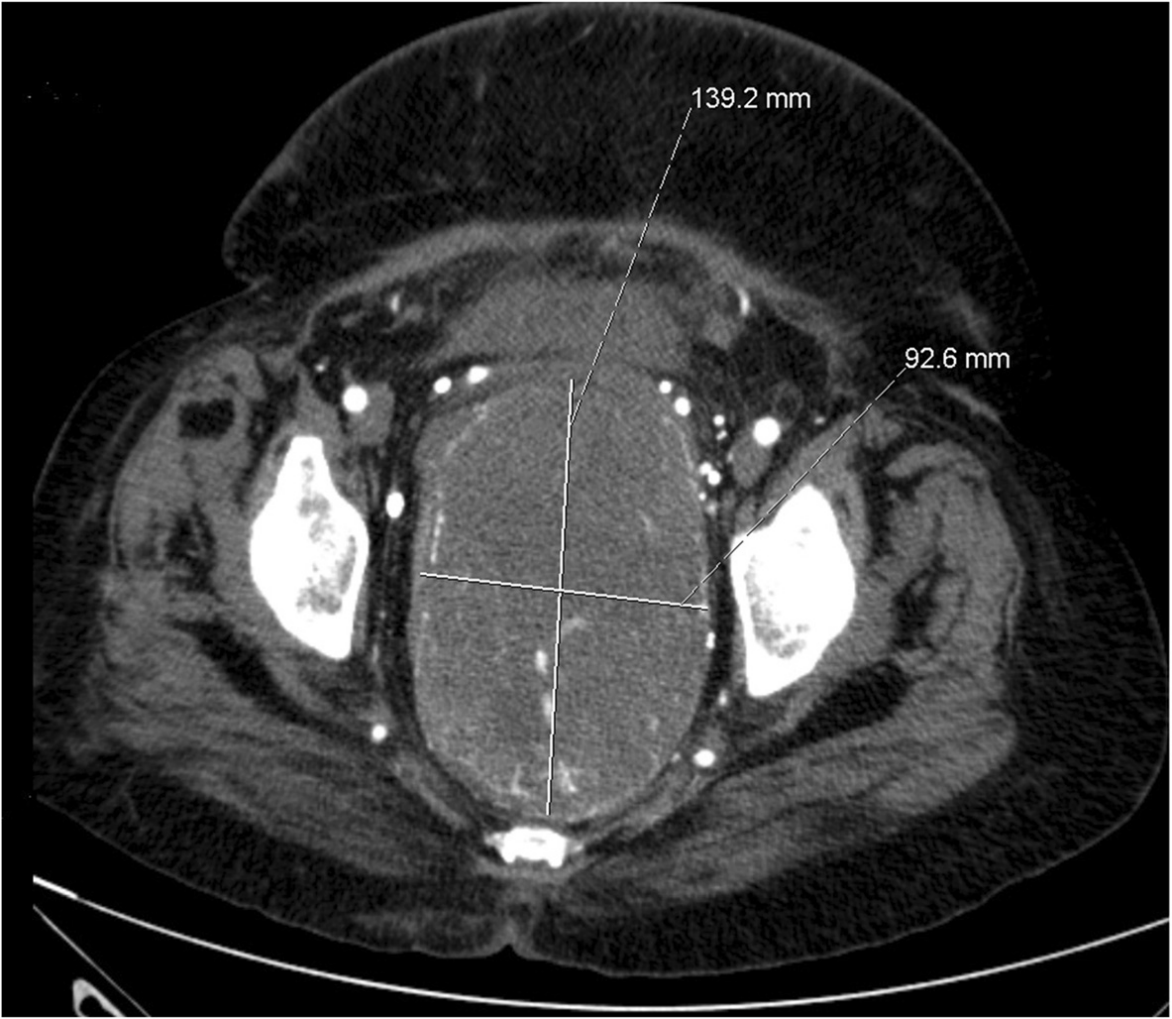
Preoperative CT showing the size, vascularization, and location of the tumor

A 70-year-old female patient was presented with lower abdominal pain and constipation at our hospital. A computed tomography (CT) was performed and revealed a highly vascularized solid mass measuring 9 × 14 × 19 cm, congestion of the kidneys, and compression of the rectum (Fig 1). The highly vascularisation raised concerns of high blood loss during surgery, and therefore, we decided at our interdisciplinary tumor conference (multidisciplinary team (MDT)) first, to perform a preoperative arterial embolization and resect the mass subsequently.

**Fig. 2:**
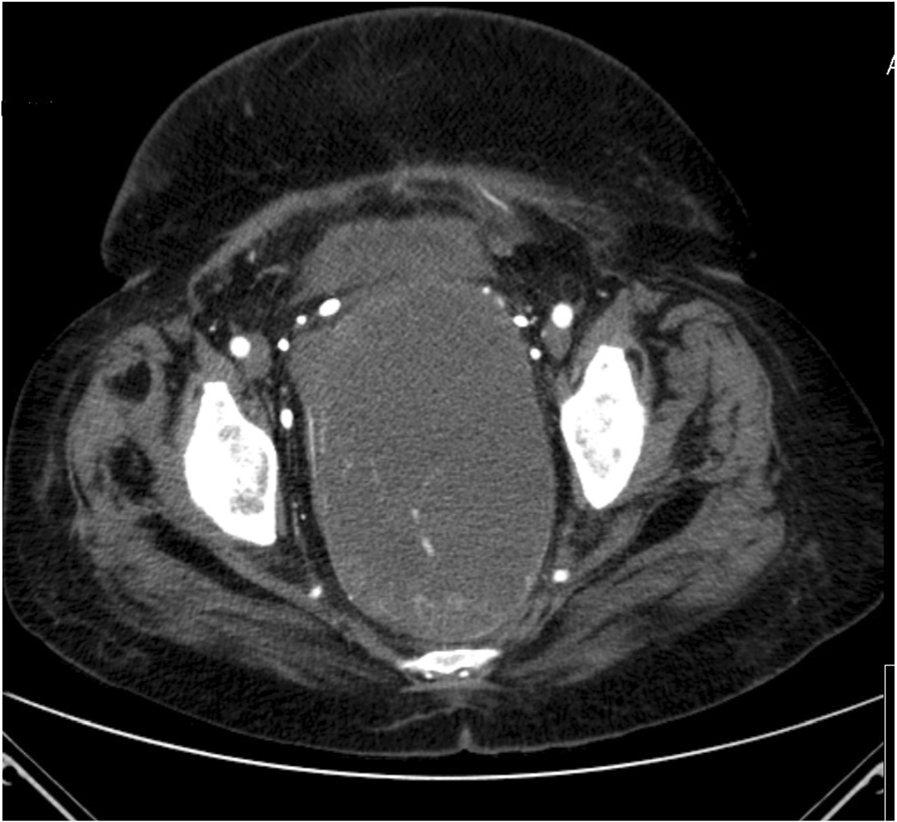
Preoperative and postembolization CT showing partial devascularization

**Fig. 3:**
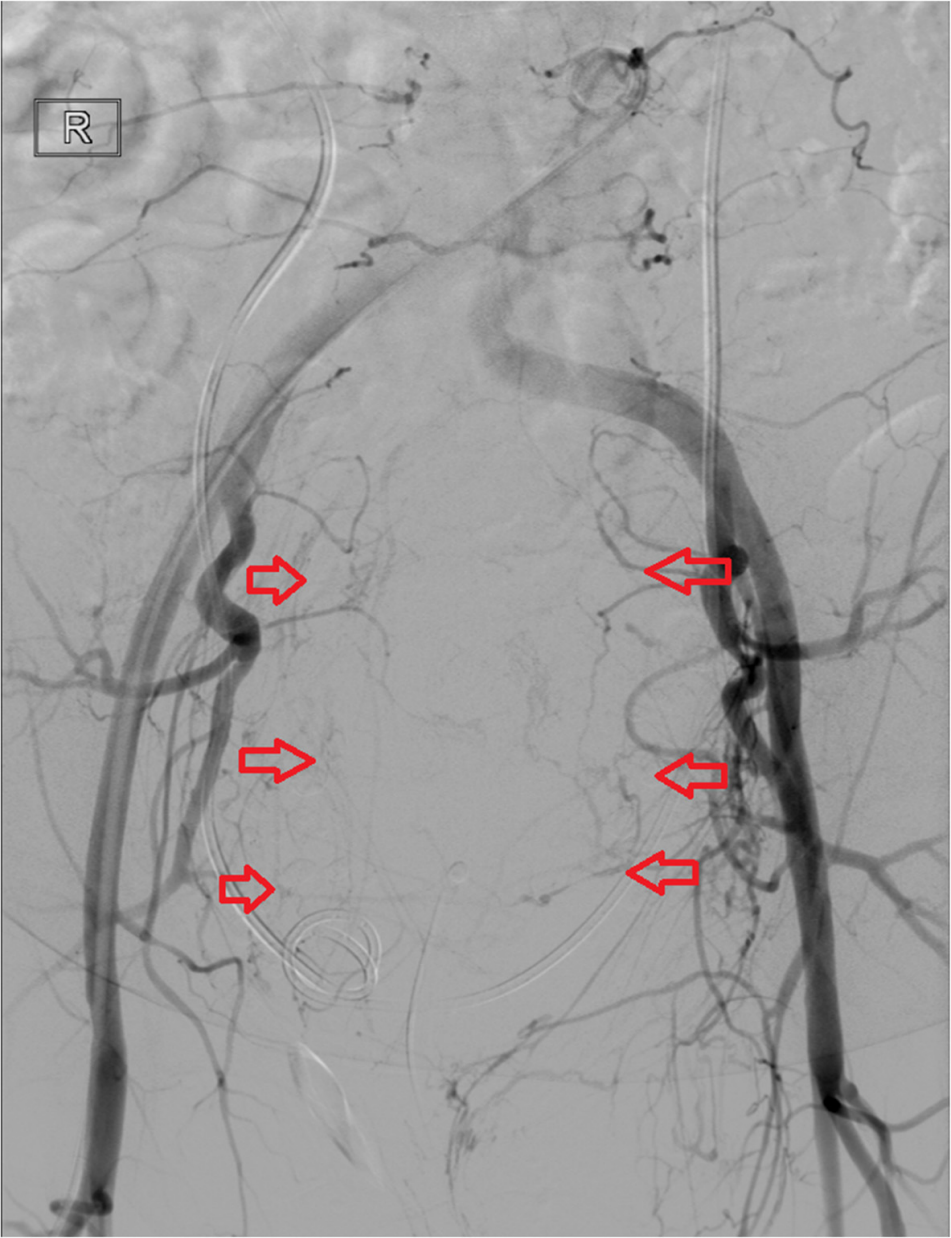
Digital subtraction angiography showing the vascularization of the tumor in the pelvis (arrows indicate the vasculature)

**Fig. 4:**
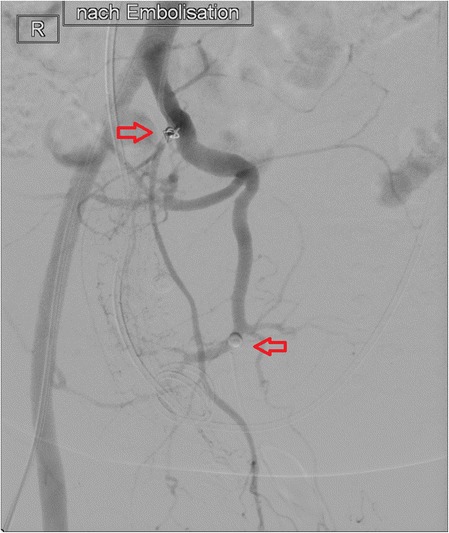
Digital subtraction angiography after the embolization (*arrows* indicate the coils)

An uneventful preoperative arterial embolization of the superior and medial rectal arteries with coils and polyvinyl alcohol particles was performed. The intervention was done without any complications, and the patient recovered fast. Two weeks after the embolization, we obtained a CT to assess the tumor, regarding size and blood supply. As shown in Fig. 2, there was a partial devascularization but unchanged size (Fig. 2). Figure 3 shows a digital subtraction angiography (DSA) with a vascularization of the tumor prior to embolization and Fig. 4 a DSA after the embolization with the coils (Figs. 3 and 4)

**Fig. 5:**
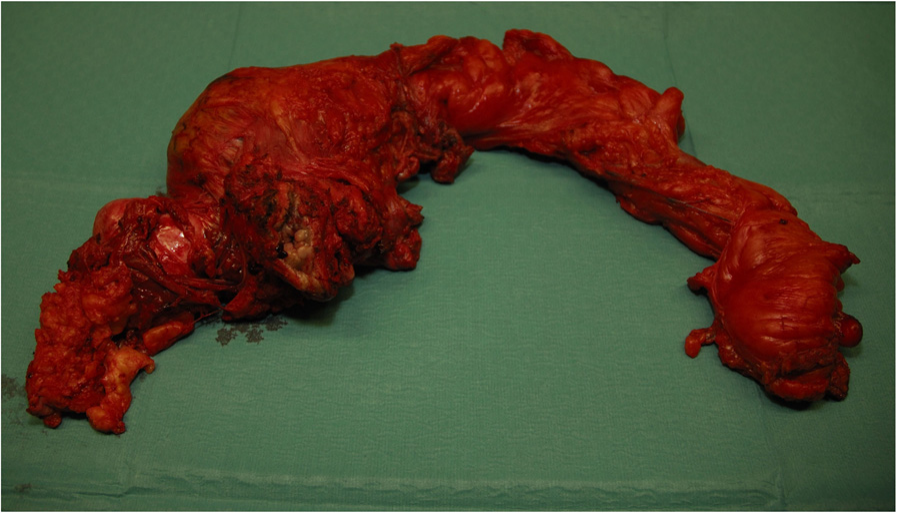
Specimen ex-situ

Prior to the operation, transureteric stents were placed. We took a standard fashioned transabdominal approach to the abdomen. The large tumor was infiltrating the rectum, ovaries uterus, and vagina. An abdominoperineal extirpation was performed, and due to the massive infiltration and expulsion, an intralesional transection was done. After retrieving the tumor and securing free margins, a permanent colostomy was established (Fig. 5). The bladder was spared. The total blood loss peroperatively was less than 200 cc.

The histological finding verified a HPC with a mitotic index of 12 per 10 high-power field. Due to the high probability of local recurrence, we suggested a close monitoring with radiographic assessment. The patient recovered quickly from surgery. A delay of the discharge was due to an uncomplicated wound healing disturbance.

**Fig. 6:**
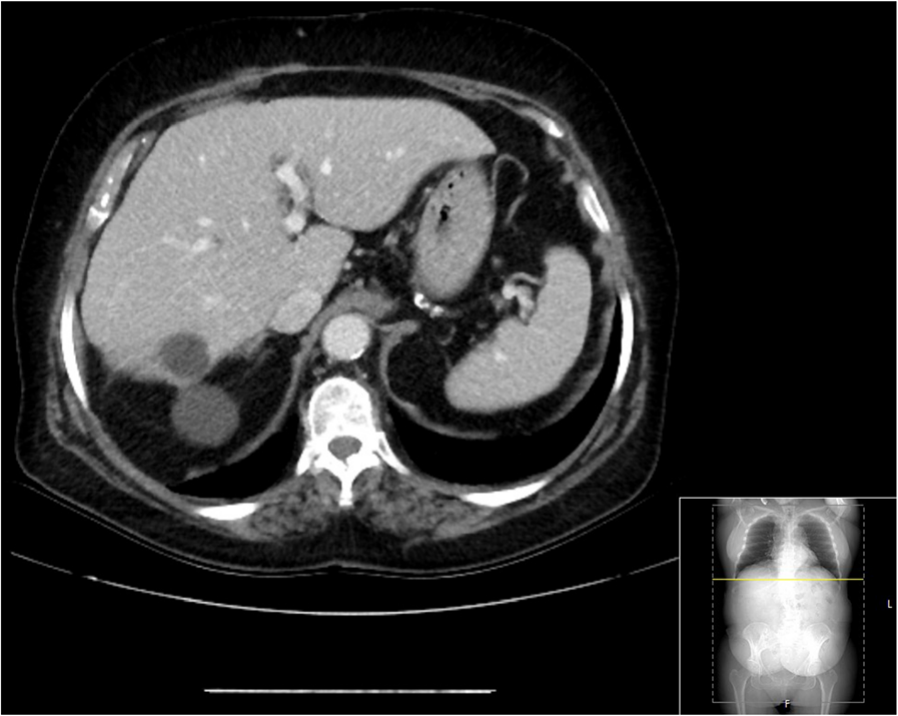
Preoperative CT before the second operation, showing the hepatic mass in segment 7 in the axial plane

**Fig. 7:**
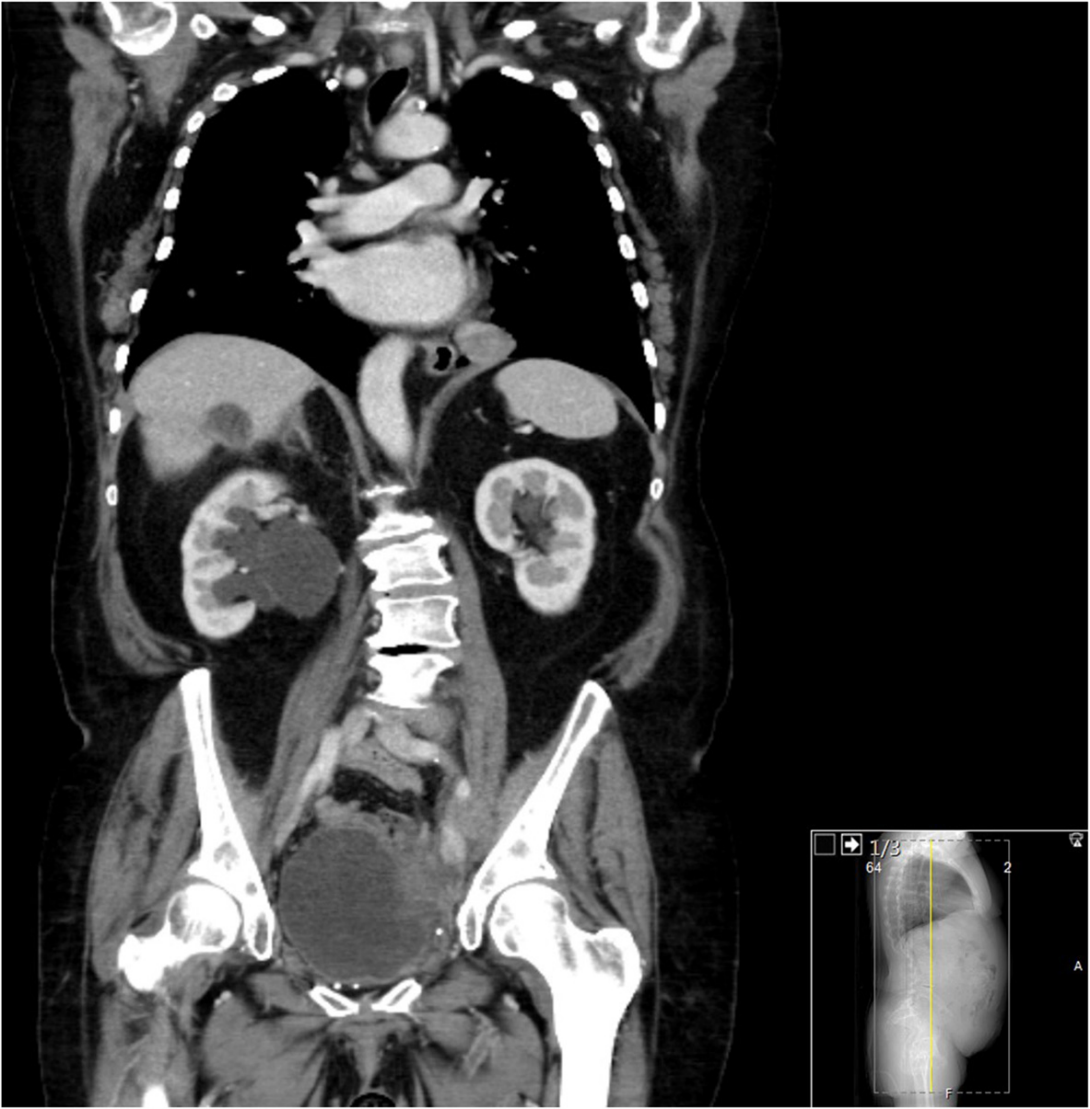
Preoperative CT before the second operation, showing the hepatic mass in segment 7 in the coronar plane

Radiological assessment after 17 months revealed a hepatic mass in segment 7 (8 cm) and a mass at the roof of the bladder (3 cm) as a sign of local recurrence. Figure 6 shows an axial CT before the operation and Fig. 7 the coronar plane of the CT (Figs. 6 and 7). Therefore, we discussed the case in the MDT and proposed a hepatic resection and local resection without prior embolization. Intraoperatively, the mass was confirmed by ultrasound, and an anatomical resection of segment 7 and partial resection of the bladder was performed. The blood loss was minimal, and the postoperative course was uneventful. The patient was discharged after 7 days. Histology revealed the solid fibrous tumor with a mitotic index of 11 per high-power field. After the second operation, the patient is without any recurrence after 13 months.

### Conclusions

Hemangiopericytomas are highly vascularized large tumors, which arise from pericytes, and are found more commonly in the abdomen or pelvis [7–12]. There are cases which describe tumors in the mediastinum, esophagus, and femur. Even peritoneal carcinomatosis has been seen [5, 13–15]. The symptoms vary from bleeding, extrusion, or pain [16, 17]. HPCs, especially those located in the abdomen, can be as aggressive as angiosarcomas, and metastasis to the bone and lung has also reported [13, 18]. As described before, the potential life threatening blood loss poses a difficult issue for the surgeon. Complete resections of these tumors are often limited due to the fact that viable structures are affected. We believe that preoperative angiographic imaging and subsequent embolization are crucial to guarantee complete resection and concomitantly reduce peroperative morbidity and mortality. The possibility to achieve a complete debulking with free margins can be reached with this approach. In 2012, Asano et al. published a similar case in which prior to the removal of a large HPC an embolization was performed with a good outcome [19].

Do we change the rate of recurrent disease if we perform a transection of these large tumors, as done in this case? Although, data presented by Espat et al. in 2002 showed with a collective consisting of 25 patients with a follow-up of 49 months metastases in 20 % and local recurrence in 4 % of the resected patients, interestingly, our patient did recur locally and distantly [20]. An effect of the transection cannot be proven, but one must be cautious if transection is performed. The postoperative course after the second operation was uneventful and blood loss minimal. One may ask, why we did not perform embolization of the tumors before the second operation? We think that, unlike highly vascularized, not easily accessible and bulky tumors of the pelvis (like in this case) in liver surgery vascular control is mandatory and inherently part of hepatic resection. Certainly, also the size and the location of the tumor in the liver are vital for the decision to perform transarterial embolization. The size and the location of the hepatic metastases in this case were favorable (Fig. 6). Additionally, the size of the local recurrence was only 3 cm and easily accessible.

In 2015, Manatakis et al. described a case of hepatic metastases of a HPC. In their final statement, they proposed transarterial embolization due to the “hypervascularity of the tumor”. If the case is read carefully, the authors describe a first liver resection without (!) embolization with an uneventful course and state that before the second liver resection, the transarterial embolization was used as tool to “bridge” [21]. Also in 2015, Vetorazzo et al. described a case of a renal HPC without prior embolization with an uneventful resection [22].

Bokshan et al. showed that even larger resections could be safely performed if in the hands of a dedicated center. In 2012, they published a case of a hepatic metastasized HPC and an ex vivo resection with veno-venous bypass, and reconstruction of the left liver vein with replacement of the inferior vena cava was performed without mortality [23].

Table 1 illustrates recently published case reports describing HPCs of the abdomen (Table 1)

**Table 1:** List of recently published case reports with HPCs in the abdomen

Author	Year	Localization	Embolization	Morbidity	Mortality	Follow-up (in months)
Manatakis et al.	2015	Liver (met^b^)	No	None	None	N.a
Vetorazzo et al.	2015	Kidney (pri^a^)	No	None	None	7
Hu et al. [24]	2014	Kidney (pri)	No	None	None	12
Bokshan et al.	2012	Liver (met)	No	None	None	N.a.
Asano et al.	2012	Pelivs (pri)	Yes	None	None	3
Katsuno et al.[25]	2011	Pelvis (pri)	No	None	None	24

Follow-up indicates time of recurrent-free-survival

^a^Primary

^b^Metastasis

Due to the low-incidence of HPCs in the abdomen, a clear statement regarding a preoperative embolization (which localization? which size? or what timing?) cannot be given. We propose following approach for tumors affecting visceral organs in the abdominal cavity:

If the tumor is located in the abdominal cavity/pelvis and is large (a clear value cannot be given but large in the sense of infiltrating or displacing other organs), an embolization needs to be performed. On the other hand, if the location and the size of the tumor are favorable, a hepatic resection can be performed straightaway.

Certainly, the most crucial part of the treatment is a thorough discussion in the MDT.

In our opinion, preoperative embolization should be incorporated into this interdisciplinary approach.

## Consent

Written informed consent was obtained from the patient for publication of this case report and any accompanying images. A copy of the written consent is available for review by the Editor-in-Chief of this journal.
